# Coordinated expression and genetic polymorphisms in Grainyhead-like genes in human non-melanoma skin cancers

**DOI:** 10.1186/s12885-017-3943-8

**Published:** 2018-01-04

**Authors:** Agnieszka Kikulska, Tobias Rausch, Ewa Krzywinska, Magdalena Pawlak, Bartek Wilczynski, Vladimir Benes, Piotr Rutkowski, Tomasz Wilanowski

**Affiliations:** 1 Department of Cell Biology, Laboratory of Signal Transduction, Nencki Institute of Experimental Biology of Polish Academy of Sciences, 3 Pasteur St, 02-093 Warsaw, Poland; 2 Genomics Core Facility, European Molecular Biology Laboratory, Meyerhofstraβe 1, 69117 Heidelberg, Germany; 3 Computational Biology Group, Institute of Informatics, University of Warsaw, 2 Banacha St, 02-097 Warsaw, Poland; 4 Department of Soft Tissue/Bone Sarcoma and Melanoma, Maria Sklodowska-Curie Memorial Cancer Center and Institute of Oncology, 5 Roentgena St, 02-781 Warsaw, Poland

**Keywords:** Non-melanoma skin cancer, Molecular genetics, Gene expression, microRNA, Single nucleotide polymorphism, Transcription factor, Grainyhead-like

## Abstract

**Background:**

The Grainyhead-like (GRHL) transcription factors have been linked to many different types of cancer. However, no previous study has attempted to investigate potential correlations in expression of different *GRHL* genes in this context. Furthermore, there is very little information concerning damaging mutations and/or single nucleotide polymorphisms in *GRHL* genes that may be linked to cancer.

**Methods:**

DNA and RNA were extracted from human non-melanoma skin cancers (NMSC) and adjacent normal tissues (*n* = 33 pairs of samples). The expression of *GRHL* genes was measured by quantitative real time PCR. Regulation of *GRHL* expression by miRNA was studied using cell transfection methods and dual-luciferase reporter system. Targeted deep sequencing of *GRHL* genes in tumor samples and control tissues were employed to search for mutations and single nucleotide polymorphisms. Single marker rs141193530 was genotyped with pyrosequencing in additional NMSC replication cohort (*n* = 176). Appropriate statistical and bioinformatic methods were used to analyze and interpret results.

**Results:**

We discovered that the expression of two genes – *GRHL1* and *GRHL3* – is reduced in a coordinated manner in tumor samples, in comparison to the control healthy skin samples obtained from the same individuals. It is possible that both *GRHL1* and *GRHL3* are regulated, at least to some extent, by different strands of the same oncogenic microRNA – miR-21, what would at least partially explain observed correlation. No de novo mutations in the *GRHL* genes were detected in the examined tumor samples. However, some single nucleotide polymorphisms in the *GRHL* genes occur at significantly altered frequencies in the examined group of NMSC patients.

**Conclusions:**

Non-melanoma skin cancer growth is accompanied by coordinated reduced expression of epidermal differentiation genes: *GRHL1* and *GRHL3*, which may be regulated by miR-21–3p and -5p, respectively. Some potentially damaging single nucleotide polymorphisms in *GRHL* genes occur with altered frequencies in NMSC patients, and they may in particular impair the expression of *GRHL3* gene or functioning of encoded protein. The presence of these polymorphisms may indicate an increased risk of NMSC development in affected people.

**Electronic supplementary material:**

The online version of this article (10.1186/s12885-017-3943-8) contains supplementary material, which is available to authorized users.

## Background

Redundancy is recognized as one of the greatest challenges in developing new approaches to combat cancer [1]. One example of redundancy concerns enzyme function, where enzymes encoded by different genes can catalyze the same chemical reaction. Another example of such phenomenon is when different transcription factors can regulate the expression of common target genes.

In mammals, proper structure and regeneration of various epithelia are dependent on three members of the Grainyhead-like (GRHL) family of transcription factors, which are currently termed GRHL1, GRHL2 and GRHL3. It was already shown that there are many genetic interactions between different *Grhl* genes. The best studied example concerns the roles of *Grhl2* and *Grhl3* in neural tube closure in mouse models [2]. In that case, the two genes exhibit partially redundant roles, without being fully functionally equivalent, what is explained by partially overlapping target gene specificity, as GRHL2 and GRHL3 share some of their target genes, while other target genes are unique to each factor [3].

In the maintenance of adult skin barrier, genetic redundancy involves the *Grhl1* and *Grhl3* genes, which was recently demonstrated using mouse models. The epidermis of *Grhl1*-null mice is impermeable to extrinsic dyes and these mice are viable [4]. Similarly, *Grhl3* conditional knockout mice, in which the *Grhl3* gene has been selectively inactivated in the epidermis after birth, display no apparent skin barrier defects and survive well into adulthood [5]. However, when both *Grhl1* and *Grhl3* genes are simultaneously inactivated in the epidermis in adult mice, this leads to a complete loss of barrier impermeability which is lethal [6]. This phenotype can be explained by the regulation of expression of different cross-linking enzymes by different GRHL transcription factors.

Transglutaminases (TGM) are enzymes which catalyze the formation of extensively cross-linked, insoluble protein polymers to establish the epidermal cornified envelope [7]. In the mouse epidermis, GRHL3 regulates the expression of *Tgm1* while GRHL1 regulates the expression of *Tgm5* and, to a lesser extent, *Tgm1*. Consequently, in *Grhl1*-null mice and *Grhl3* conditional knockout mice the total transglutaminase activity remains sufficiently high to ensure impermeability of skin barrier. However, concomitant loss of both *Grhl1* and *Grhl3* in the epidermis results in greatly reduced levels of both TGM1 and TGM5, markedly reduced total transglutaminase activity and loss of skin barrier impermeability which is incompatible with life [6].

All of the GRHLs have been implicated in various types of cancer (these findings have been summarized in a recent review [8]). Two of them, GRHL1 and GRHL3, have been linked to the development of cutaneous carcinoma of the skin. When subjected to the standard chemical skin carcinogenesis protocol, the *Grhl1*-null mice develop more squamous cell carcinomas (SCC), with an earlier onset, than their control wildtype littermates [9]. Similar phenotype has been observed in conditional *Grhl3* knockout mice with epidermis-specific ablation of *Grhl3* after birth [5]. However, the underlying molecular mechanisms are different: in the *Grhl3* conditional knockout epidermis, there is a reduction in the level of tumor suppressor phosphatase and tensin homolog (PTEN), a direct target of GRHL3 regulation, which triggers dysregulation of the PI3K/AKT/mTOR signaling pathway [5]; in the *Grhl1*^−/−^ mice, the underlying molecular mechanism involves aberrant keratinocyte terminal differentiation and subacute skin barrier defects which, by inducing tumor-promoting mild chronic inflammatory microenvironment in the skin, increase the risk of skin cancer [9, 10].

On the basis of the latest discoveries that: *(i)* the role of barrier genes in cutaneous carcinoma formation is well documented [11]; *(ii)* the *Grhl* genes are important for the epidermal barrier maintenance and they display a degree of redundancy in this context [6]; *(iii)* both *Grhl1* and *Grhl3* serve protective roles against the development of SCC of the skin in mouse models [5, 9]; we hypothesize that simultaneous downregulation of different *GRHL* genes, whose genetic redundancy and genetic interactions are evident, is relevant in skin carcinoma formation. Reduced expression of *GRHL3* in human basal cell carcinoma (BCC) and squamous cell carcinoma (SCC) of the skin has already been observed, but other *GRHL* genes were not investigated in those studies [5, 12]. To test this hypothesis we assayed the expression of all the *GRHL1–3* genes in two subtypes of human non-melanoma skin cancer samples – BCCs and SCCs. We also looked for common regulatory factors (such as microRNA) that may affect *GRHLs* expression. Furthermore, we searched for damaging mutations in all the *GRHL1–3* genes in NMSC samples and in control healthy tissues from the same patients. In addition, we looked for single nucleotide polymorphisms in *GRHL* genes that might predispose human subjects to NMSC.

## Methods

### Patient cohort and sample collection

Thirty-three Polish patients with NMSC were enrolled in this study (22 with BCC and 11 with SCC). The patients were surgically treated in the Department of Soft Tissue/Bone Sarcoma and Melanoma in the Maria Sklodowska-Curie Memorial Cancer Center and Institute of Oncology in Warsaw, Poland. The study was approved by local Bioethical Committee (permit number 13/2008). After surgical resection of an entire diseased area with a margin, small tissue samples were resected from both: the core of lesion and control normal skin from the border of excision and stored at −80 °C until use. General information about the examined group of patients and histopathological classification of carcinomas is provided in Additional file 1: Table S1.

### Expression of *GRHL* genes in cancer samples

#### RNA extraction

Total RNA was extracted from all collected fresh tissues (carcinoma and normal tissue from the border of excision) using RNeasy® Fibrous Tissue Mini Kit (Qiagen, cat. no. 74704) according to manufacturer’s instructions. The purity of RNA was determined using NanoDrop 2000 UV–vis spectrophotometer (Thermo Fisher Scientific). Assessment of RNA quality was performed with 2100 Bioanalyzer instrument and RNA 6000 Nano Kit (Agilent Technologies). Good quality samples (*n* = 27 pairs, cancer and normal tissue from the same patient) with RNA Integrity Number (RIN) higher than 5 were included in further analyses. RNA concentration was determined using the Qubit® 2.0 Fluorimeter and RNA BR Assay (Thermo Fisher Scientific, cat. no. Q10210).

#### Reverse transcription and real-time PCR

cDNA was synthesized from 250 ng of total RNA with SuperScript® VILO™ Master Mix (Invitrogen, cat. no. 11755050). The levels of expression of *GRHL* genes were assayed using Applied Biosystems chemistry: TaqMan® Fast Universal PCR Master Mix No AmpErase UNG (cat. no. 4352042) and TaqMan Gene Expression Assays (Assay ID: Hs01119372_m1 for *GRHL1*, Hs00227745_m1 for *GRHL2*, Hs00297962_m1 for *GRHL3*, Hs03929098_m1 for *HPRT1* control). Real-time quantitative PCR was performed in 7900HT Fast Real-Time PCR System (Applied Biosystems). Gene expression was normalized to *HPRT1* housekeeping gene and statistical differences were determined for relative expressions (2^-∆∆Ct^) with two-tailed Mann–Whitney U test with significance level < 0.05.

### Interaction of miR-21–3p with 3′ untranslated region (UTR) of *GRHL1*

#### Cell culture

HaCaT cell line was bought from Cell Lines Service (cat. no. 300493). HEK293T cells were a kind gift from Ewelina Szymanska; the origin of this cell line is explained in a recent review article [13]. HaCaT and HEK293T cells were routinely cultured in DMEM GlutaMAX medium (Thermo Fisher Scientific, cat. no. 10566–016) supplemented with 10% fetal bovine serum (Thermo Fisher Scientific, cat. no. 10270–106) and 100 IU/mL penicillin-streptomycin (Thermo Fisher Scientific, cat. no. 15140122) in a humidified incubator in an atmosphere of 95% air and 5% CO_2_ at 37 °C.

#### HaCaT cells treated with miR-21–3p mimic or hairpin inhibitor

60 nM miR-21–3p mimic or negative control (Ambion, mirVana cat. no. MC12979 and cat. No. 4464058) or 180 nM miR-21–3p hairpin inhibitor or negative control (Dharmacon, miRIDIAN cat. no. IH-301023-02 and IN-001005-01) were transfected into HaCaT cells using Lipofectamine 2000 or Lipofectamine 3000 transfection reagent (Invitrogen, cat. no. 11668019 or L3000–008). After 24 h cells were harvested with RNeasy Mini Kit (Qiagen, cat. no. 74104) or lysed in lysis buffer (50 mM Tris–HCl, pH 7.4, 150 mM NaCl, 1 mM EDTA, 1% Triton X-100 and 1× Complete™ Protease Inhibitor Cocktail from Roche) for Western blot analysis. cDNA was synthesized using High Capacity cDNA Reverse Transcription Kit (Applied Biosystems, cat. no. 4368814). Real Time PCR was carried out with TaqMan Probes (Assay ID: Hs01119372_m1 for *GRHL1*, Hs00227745_m1 for *GRHL2*, Hs00297962_m1 for *GRHL3*, Hs00427620_m1 for *TBP* and Hs99999901_s1 for 18S). Histone deacetylase 8 – *HDAC8* (Assay ID: Hs00954359_m1), which expression is regulated by miR-21–3p [14], was used as a control target. Each transfection was performed in duplicates and repeated three times. Gene expression was normalized to *TBP* housekeeping gene and statistical differences for relative expression (2^-∆∆Ct^) were determined with two-tailed Student’s t-test. For Western blot analysis 20 μg of total protein was separated on 12% SDS-PAGE gels and subsequently transferred to PVDF membrane. Membranes were blocked with 5% non-fat milk and incubated with primary antibody in blocking buffer. The following antibodies were used for immunoblotting: anti-GRHL1 (Sigma, cat. no. HPA005798), anti-rabbit IgG, HRP-linked (Cell Signaling, cat. no. 7074) and anti-β-actin (Sigma, cat. no. A3854). Quantification of protein abundance was carried out by ImageJ software and the relevant bands were normalized against the corresponding β-actin levels. Statistical analysis was performed using Student’s t-test.

#### Luciferase 3’UTR reporter assay in HEK293T cells

HEK293T cells were plated in 24-well plates and transiently transfected with 50 ng of GRHL1_3’UTR, GRHL1_3’UTR_mut or negative control vector (GeneCopoeia, cat. no. HmiT055586-MT01 and cat. no. CmiT000001-MT01,) using Lipofectamine 2000 (Invitrogen, cat. no. 11668019) following the manufacturer’s protocol. At the same time, miR-21–3p mimics or negative control were co-transfected with reporter vector in a final concentration of 60 nM. The 3’UTR of human *GRHL1* was mutated using a QuikChange Site-Directed Mutagenesis Kit (Agilent Technologies, cat. no. 200518). Cells were harvested 24 h after transfection using the reporter lysis buffer (Promega). Firefly and *Renilla* luciferase activities were analyzed at room temperature in a multimode reader Infinite M1000Pro (Tecan) using the Dual-Luciferase Reporter Assay System (Promega, cat. No. E1910). Relative luciferase activity was defined as the mean value of the firefly/*Renilla* normalized ratios obtained from 3 independent biological replicates. Statistical differences were indicated with 2-tailed Student’s *t-*test.

### Expression of miR-21–3p and *GRHL1* in cancer cell lines

Squamous cell carcinoma cell lines were purchased from the American Type Culture Collection: A-431 (cat. no. CRL-1555), CAL-27 (cat. no. CRL-2095), SCC-15 (cat. no. CRL-1623), SCC-25 (cat. no. CRL-1628). HaCaT cell line was bought from Cell Lines Service (cat. no. 300493). SCC-351 cell line was a kind gift from Agnieszka Kobielak; this cell line is also known as USC-HN1 and originates from the laboratory of Alan L. Epstein, Department of Pathology, Keck School of Medicine of the University of Southern California, Los Angeles, CA, USA, and was first described by the members of his laboratory [15]. All cell lines were cultured as described above for the HaCaT cell line. RNA extraction, cDNA synthesis and TaqMan assays were carried out as described above.

### Mutations and polymorphisms in *GRHL* genes

#### DNA extraction, target enrichment and next generation sequencing

DNA from 10 to 15 mg of homogenized tissues (Bio-Gen PRO homogenizer) was extracted using QIAamp® DNA Mini Kit (Qiagen, cat. no. 51304) according to manufacturer’s instructions. The purity of DNA was determined with the NanoDrop 2000 UV–vis spectrophotometer (Thermo Fisher Scientific). Accurate DNA concentration was quantitated using the Qubit® 2.0 Fluorimeter and dsDNA BR Assay (Invitrogen; cat. no. Q32853). SureDesign HaloPlex Standard Wizard was employed to select the custom probe sequences based on target regions of *GRHL* genes, according to the hg19/GRCh37 assembly from UCSC database ([16] Feb, 2009 version); the list of analyzable regions is provided in Additional file 1: Table S2. Capture of the targeted regions was performed with reagent set from a custom design HaloPlex Target Enrichment System 1–500 kb (Agilent Technologies), according to the Protocol Version D (August 2012). Briefly, the protocol consisted of the four following steps: 1) digestion of genomic DNA (250 ng) by restriction enzymes in eight parallel reactions; 2) hybridization resulting in circularization of digested DNA fragments with complementary probes which incorporated indexes and Illumina sequencing motifs; 3) capture of targeted DNA using streptavidin beads and ligation of circularized fragments; 4) PCR amplification of captured target libraries. Paired-end sequencing of samples was performed on a MiSeq instrument (Illumina) in Genomics Core Facility, European Molecular Biology Laboratory, Heidelberg, Germany.

#### NGS data processing

##### Data preprocessing

Sequence reads were resynchronized and trimmed to remove Illumina adapter sequences and only reads longer than 36 bp were kept. Sequences were further filtered with Trimmomatic [17] for low quality leading/trailing bases with phred quality lower than 20. Subsequently sequences were aligned to the human reference genome (version hg19) with Stampy [18]. Additionally, the initial 5 bases were trimmed due to potential allele bias in case of single nucleotide polymorphisms (SNP) present in restriction enzyme cutting sites. SNPs were called with SAMtools mpileup algorithm with default parameters [19]. Coverage cutoff value was 20.

##### SNPs of association

Distribution of SNPs in examined NMSC population was compared to the European population (data derived from 1000Genomes database [20]) and association *p-*value was determined with a χ^2^ – test or Fisher’s exact test. The *p*-value threshold for significance was adjusted with multiple-comparison correction (Bonferroni correction).

##### Predicted effects of SNPs in TF binding motifs

Mapping of SNPs to Encyclopedia of DNA Elements Consortium (ENCODE) regions [21] and transcription factor binding motifs was performed with the Nencki Genomics Database-Ensembl funcgen [22]. The motif matches against DNA sequences were scored as the log-odds of the respective position weight matrix of the motif derived from the JASPAR database [23]. For each SNP, the original reference sequence and the sequence modified by a single SNP were considered (interactions of multiple SNPs in the same motif were not considered). The differences between the log-odds scores were interpreted as the logarithm of the fold change of the binding energy. In cases where the odd scores of the mutated sequence were 0 (no motif match at all); the log odds differences were interpreted as infinite (log(x) approaches negative infinity as x approaches 0).

### Genotyping of exonic SNPs rs141193530 and rs41268753 in a replication cohort

#### Replication samples

Formalin-fixed, paraffin-embedded (FFPE) materials from 177 Polish patients with non-melanoma skin cancers (144 with BCC and 32 with SCC) were randomly selected. The patients were surgically treated in the Department of Soft Tissue/Bone Sarcoma and Melanoma in the Maria Sklodowska-Curie Memorial Cancer Center and Institute of Oncology, Warsaw, Poland and the study was approved by the Bioethical Committee; permit number 13/2008.

#### DNA extraction

DNA extraction from FFPE samples (5–10 slices of 10-μm-thick sections) was performed using NucleoSpin DNA FFPE XS (Macherey-Nagel), all steps were performed according to manufacturer’s instructions. DNA quantitation was carried out with Qubit™ dsDNA HS Assay Kit (Invitrogen, cat. no. Q32854). To obtain useful information, including high-quality sequence data, FFPE DNA samples were treated with NEBNext FFPE DNA Repair Mix (New England BioLabs, cat. no. M6630 L) according to manufacturer’s instructions.

#### PCR-RFLP

To identify DNA samples with potential SNPs in their sequences, preliminary selection was performed with PCR-RFLP (restriction fragment length polymorphism). PCR amplification of genomic DNA was conducted using forward primer: 5’–CTTCAGGGGCAATGAGACGAC–3′ and reverse primer 5’–GCACATTGGGGATGAACAGC–3′, the annealing temperature for PCR reactions was 65 °C. For accurate replication of template, Q5® High-Fidelity DNA Polymerase was used (NEB, cat. no. M0492S) and PCR products (size 80 bp) were digested with *Bsa*HI restriction enzyme (NEB, cat. no. R0556S), which digests only templates without examined SNPs (the presence of either rs141193530 but also rs41268753 will abolish the restriction site 5’-GRCGYC-3′). The digested PCR products were resolved in 2.5–3% High Resolution Agarose (EurX, Poland, cat. no. E0302–50) gels, stained with SimplySafe™ (EurX, Poland, cat. no. E4600–01) and visualized with G:Box (Syngene). The digested PCR product of each sample was compared to the same amount of non-digested product. Samples with positive outcome (product not digested or partially digested) were additionally genotyped by pyrosequencing.

#### Pyrosequencing

To specify exact single nucleotide polymorphism in selected DNA samples, a pyrosequencing assay was designed to measure the relative quantification of nucleotide incorporation at the SNP sites: rs41268753 (C/T) and rs141193530 (C/G). PCR amplification primers for the genomic DNA templates were as follows: Fw_5’–biotinylated–CTTCAGGGGCAATGAGACGAC–3′ and Rv_5’–GCACATTGGGGATGAACAGC–3′, with primer annealing at 65 °C. The biotinylated PCR products (80 bp) were subject to pyrosequencing using internal pyrosequencing primer 5’-ATTGGGGATGAACAGCAC–3′. The sequence content analyzed was GGGTG[G/C]C[G/A]TCTCC. Pyrosequencing service was provided by A&A Biotechnology (Gdynia, Poland).

#### Statistical analysis

For analysis of single marker association, SNP frequencies in coding regions in control European non-Finnish population were obtained from the Exome Aggregation Consortium database (ExAC) [24]. For calculating odds ratio, relative risk, confidence interval, significance level and other parameters, statistical methods appropriate for medical research were employed [25]. General information about the considered group of patients is provided in Additional file 1: Table S3.

## Results

### Reduced expression of *GRHL1* and *GRHL3* genes in human non-melanoma skin cancers

**Fig. 1:**
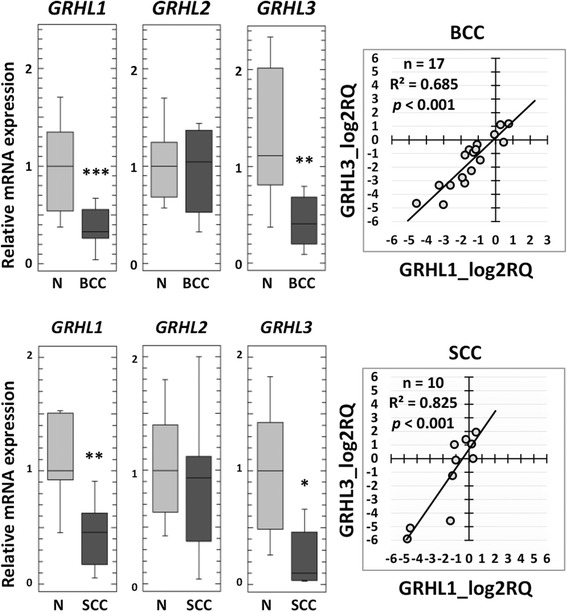
Expression of *GRHL* genes in non-melanoma skin cancer samples. cDNA pools from 27 samples of NMSC (17 BCC and 10 SCC) were analyzed; gene expression was normalized to *HPRT1*. On the left: box plot representations of 2^-∆∆Ct^ in subtypes of NMSC [39]**.** Each box indicates the 25th and 75th percentiles of the distribution. The horizontal line inside the box indicates the median, and the whiskers indicate the extreme measured values. *P*-values were derived from Mann–Whitney U test. On the right: correlation plots of *GRHL1* and *GRHL3* expression. BCC – basal cell carcinoma, SCC – squamous cell carcinoma, N – normal skin from the border of excision, R^2^ – Spearman correlation coefficient

We analyzed the expression of *GRHL1–3* genes by Real-Time PCR in *n* = 27 NMSC samples (17 BCCs and 10 SCCs), including tumors and the adjacent histologically normal tissue from the border of excision. Expression levels of two of the examined genes – *GRHL1* and *GRHL3* – were significantly reduced in basal cell carcinoma (BCC) samples as well as in squamous cell carcinoma (SCC) samples, in comparison with the control healthy tissue from the same patient. We did not detect any significant changes in *GRHL2* expression in either BCC or SCC cases. Interestingly, in both BCC and SCC, we observed statistically significant correlation between the expression of *GRHL1* and *GRHL3* genes, with the Spearman correlation coefficient R^2^ = 0.685 for BCC and 0.825 for SCC, with *p* < 0.001 for both (Fig. 1).

### miR-21–3p may directly target *GRHL1* by interaction with its 3’UTR

**Fig. 2:**
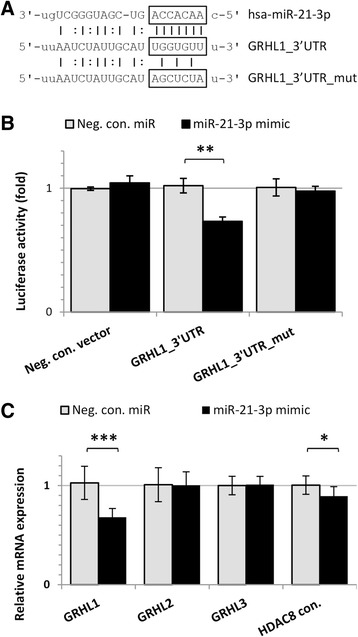
miR-21–3p may directly target *GRHL1* by interacting with its 3’UTR. **a** Schematic representation of the predicted target site of miR-21–3p in the 3’UTR of *GRHL1*. **b** Firefly*/Renilla* luciferases activity was analyzed in HEK293T cells 24 h after transfection with indicated plasmids and miR-21–3p or negative control miR, *n* = 3 biological replicates, *P < 0.05, **P < 0.01, data are shown as mean ± SD. C) Expression of *GRHL* genes and *HDAC8* control gene measured by real-time PCR after treatment of HaCaT cells with miR-21–3p mimic (c = 60 nM; experiment was performed three times in duplicates). *HDAC8* served as a positive control, because its expression is inversely regulated by miR-21–3p, therefore the level of *HDAC8* mRNA is expected to decrease following cell treatment with miR-21–3p mimic [14]. *TBP* was used as a reference gene and Student *t*-test was used to determine statistical significance. Data are shown as mean ± SD

Coordinated decrease in the levels of expression of *GRHL1* and *GRHL3* in NMSC samples may indicate a common mechanism of regulation. We decided to look for miRNAs that may target mRNAs of both *GRHL1* and *GRHL3* using target microRNA and TargetScanHuman prediction algorithms [26, 27]. Multiple sequence alignment detected seed sequences with good SVR score for miR-21–3p in the 3’UTR of *GRHL1* (Fig. 2a) which indicated that *GRHL1* is a potential target of miR-21–3p regulation. It was previously demonstrated that miR-21-5p regulates the expression of *GRHL3* [5] hence in the present study we focused on the regulation of *GRHL1* by miR-21–3p. When pMT01-GRHL1_3’UTR was co-transfected with miR-21–3p mimic into HEK293T cells, the relative luciferase activity of firefly reporter was significantly reduced compared with the transfection of negative control as well as with mutant reporter or empty vector (Fig. 2b). Furthermore, we observed decreased level of *GRHL1* mRNA in human keratinocytes treated with miR-21–3p mimic (Fig. 2c). However, the decrease of the amount of GRHL1 protein was not statistically significant (Additional file 1: Fig. S1A). We transfected HaCaT cells with a miR-21–3p inhibitor, but we did not detect a significant change in the *GRHL1* transcript or protein level, even though we tested a wide range of inhibitor concentrations, up to 180 nM (Additional file 1: Fig. S1B-C).

### Expression of miR-21–3p and *GRHL1* in SCC cell lines

**Fig. 3:**
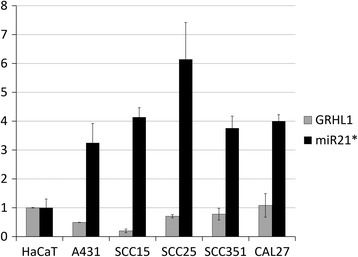
Expression of miR-21–3p and *GRHL1* in cancer cell lines relative to HaCaT cells

We assayed the expression of miR-21–3p and *GRHL1* in five SCC cell lines (Fig. 3). In all these lines we observed an increase in miR-21–3p levels, in comparison with the control HaCaT cell line. In almost all cases this increase was accompanied by a reduction in *GRHL1* expression.

### Polymorphisms in *GRHL* genes in examined cohort of patients with NMSC

We performed targeted deep sequencing in 33 human NMSC and control normal skin from the border of excision. The following regions of *GRHL* genes were sequenced: all the coding sequences, intron/exon boundaries, 5′ and 3’ UTRs, as well as all the potential regulatory regions as determined by ENCODE [21]. These included CpG islands, regions with increased sensitivity to DNase I, regions with chromatin containing histone modifications specific for open chromatin, or binding regions for general transcription factors. The full list of analyzable regions is provided in Additional file 1: Table S2. This experiment did not detect any de novo mutations in these genes, that is, mutations that would be present in a tumor sample but absent from healthy control tissue from the same individual. Subsequently we analyzed SNP distribution in the examined group of patients. To record the most strongly associated SNPs we initially compared the frequencies of single nucleotide variants present in the examined NMSC patients to allele frequencies for European population available in 1000Genomes database [20]. It was previously shown that genetic differences between extant European subpopulations are small [28], therefore European population was taken as a reference for SNP frequency comparison. SNPs with significantly altered frequencies are summarized in Table 1. In all cases the frequency of less common variant was increased in the patient cohort.

**Table 1:** Statistically significant hits from NGS of examined NMSC patients (*n* = 33)

Chr: *Gene*	dbSNP ID location	allele	AF_OBS	AF_EUR (1000G)	*p*-value^*^	OR, (95%CI), *p*-value^**^
Chr1: *GRHL3*	rs55927162 intergenic (dist = 4635)^***^	T/A	0.6667	0.21	*P* < 0.00001 (χ^2^)	(A) OR = 7.53 (95%CI:4.38–12.94) *P* < 0.0001
	rs56256719 intergenic (dist = 4627)^***^	T/A	0.697	0.24	P < 0.00001 (χ^2^)	(A) OR = 7.15 (95%CI:4.14–12.44) P < 0.0001
	rs151171718 intronic	A/T	0.0455	0.0013	P < 0.05 (F)	(T) OR = 36.05 (95%CI:3.69–351.64) *P* = 0.0020
	rs141193530 exon 11 (P455A)	C/G	0.0758	0.004	P < 0.00001 (χ^2^)	(G) OR = 20.63 (95%CI:4.81–88.38) P < 0.0001
	rs151326764 exon 16 (R573H)	G/A	0.0303	0.0026	P < 0.01 (F)	(A) OR = 11.81 (95%CI:1.63–85.26) *P* = 0.0143
Chr8: *GRHL2*	rs548650 intronic	G/A	0.0909	0.02	*P* = 0.000039 (χ^2^)	(A) OR = 5.73 (95%CI:2.10–15.62) *P* = 0.0006

*AF_OBS* observed allele frequency, *AF_EUR* expected allele frequency for general European population (1000Genomes)

^*^p-value of association calculated with χ^2^-test (χ^2^) or Fisher’s exact test (F) for less frequent alleles

^**^OR odds ratio for haplotype, 95%CI 95% confidence interval, calculated according to [25]

^***^distance upstream from transcription start site

### Single nucleotide polymorphisms in non-coding regions of *GRHL2* and *GRHL3* genes

Single nucleotide changes may contribute towards the risk of cancer development through their biological consequences. SNPs in the region upstream of the *GRHL3* gene (rs55927162 and rs56256719) may possibly affect binding of transcription factors to potential enhancers or other regulatory elements. However, our bioinformatic analyses revealed that these SNPs do not significantly alter known transcription factors binding motifs provided in the JASPAR database [23]. SNPs in intronic regions of *GRHL2* and *GRHL3* genes (rs151171718 and rs548650) may potentially affect splicing of the primary transcripts.

### Single nucleotide polymorphisms in the coding region of *GRHL3* gene

Two non-synonymous SNPs occur with significantly altered frequencies in our NMSC patient cohort: rs141193530 in exon 11 of the *GRHL3* gene, which causes P455A amino acid residue substitution, and rs151326764 in alternative exon 16 of the *GRHL3* gene, which causes R573H substitution (Table 1). We employed PROVEAN (Protein Variation Effect Analyzer) v1.1 [29] to assess whether the presence of these SNPs may affect protein function. This analysis predicted that the R573H substitution is either “neutral” (PROVEAN prediction) or “tolerated” (SIFT prediction). The P455A substitution was predicted to be either “neutral” (PROVEAN prediction) or “damaging” (SIFT prediction).

Other than introducing amino acid residue substitutions in the encoded protein, the presence of these SNPs may be damaging for the functioning of the *GRHL3* gene by affecting the processing of primary transcript. We employed two algorithms to search for exonic splicing enhancers (ESE). RESCUE-ESE [30] did not detect any potential ESEs that would overlap with the location of SNP rs141193530 or rs151326764. ESEfinder3.0 [31] predicted some changes in binding of serine and arginine rich splicing factors (SRSF) to the region containing SNP rs141193530, which were: loss of one SRSF5 potential binding site and creation of a novel SRSF2 potential binding site. However, given multiple caveats of ESEfinder algorithm [31] it is difficult to ascertain whether these changes may influence pre-mRNA processing.

Interestingly, SNP rs141193530 was reported in a recent publication describing variants of the *GRHL3* gene in patients with nonsyndromic cleft lip with/without cleft palate (nsCL/P) as well as nonsyndromic cleft palate only [32]. The authors’ analyses concluded that this variant is either “tolerated” or “benign”. We re-analyzed frequency data provided by the authors in Additional file 1: Table S3 [32] using appropriate statistical methods [25]. As reference, we took the frequency data for European non-Finnish population provided by the ExAC database [24] because genetic differences between extant European subpopulations are small with the possible exception of population isolates as observed for the Finns [28]. The frequency of rs141193530 in nsCL/P patient cohort was 0.016, compared to the control population of 0.00796. This difference in frequency was statistically significant, as indicated by OR = 2.0136; *p* = 0.0038; 95%CI: 1.2542–3.2329. Similarly, we observed increased frequency of this variant for NMSC patients (Table 2), what may indicate some effect of this single nucleotide change on *GRHL3* functioning and increased risk of disease.

**Table 2:** Single Marker Association in NMSC patients (*n* = 209) compared to different reference groups

*GRHL3*		Observed frequency (genotypes)^a^	Expected frequency (genotypes)^a^	Allelic OR (95%CI) *p*-value	Genotypic OR (C/G vs C/C) (95%CI) p-value
SNP ID	rs141193530	0.01667 (0/7/202)	ExAC database (Non-Finnish European)	OR = 2.12 (1.0006 to 4.5021) *P* = 0.0498	OR = 2.19 (1.0266 to 4.6798) *P* = 0.0426
chromosomal localization^b^	chr1:24,669,459 exon 11		0.00796 (6/519/32827)		
ref/alt	C/G		1kGP database (European)	OR = 3.41 (1.0760 to 10.8051) *P* = 0.0371	OR = 3.45 (1.0828 to 11.0017) *P* = 0.0362
consequence	p.Pro455Ala		0.00497 (0/5/1001)		

^a^genotypes GG/CG/CC

^b^position according to GRCh37/hg19 genome build

To further investigate whether this SNP is likely to be associated with NMSC occurrence, we carried out genotyping of SNP rs141193530 in a replication cohort, which included 176 additional patients with NMSC (Additional file 1: Table S3). The results are presented in Table 2.

## Discussion

Our research has demonstrated that the expression of two genes from the Grainyhead-like family – *GRHL1* and *GRHL3* – is reduced in a coordinated fashion in human NMSCs. In earlier work it was shown that the expression of *GRHL3* is regulated by miR-21-5p [5]. Here we provide evidence that the expression of *GRHL1* may be regulated by miR-21–3p, even though our findings are not entirely consistent in this regard. This mechanism is supported by the results of *GRHL1* and miR-21–3p expression studies in SCC cancer cell lines (Fig. 3) and, at the *GRHL1* transcript level as well as in luciferase reporter assays, by transfections with miR-21–3p mimic (Fig. 2). However, we did not detect a statistically significant reduction in GRHL1 protein level following cell transfection with miR-21–3p mimic (*p* = 0.1; Additional file 1: Fig. S1A). This discrepancy could be the result of differences in sensitivity between the two methods, especially as in our experiments transfection with miR-21–3p mimic induced relatively small – by about 30% – decrease in *GRHL1* transcript levels, as well as in luciferase reporter activity (Fig. 2). The interpretation of results of Western blotting is complicated further by the risk of cross-reactivity of antibodies with other proteins, as the three GRHL1–3 proteins display very high degrees of similarity, they are all expressed in HaCaT cells, and they have almost identical molecular weights so they cannot be separated by electrophoresis prior to Western blotting. Also, we did not observe changes in *GRHL1* transcript or protein levels following cell transfection with miR-21–3p inhibitor, despite using very high inhibitor concentration (Additional file 1: Fig. S1B-C). The most likely explanation is that the effect of miR-21–3p on GRHL1 expression is small, hence the treatment of cells with miR-21–3p inhibitor is insufficient to induce statistically significant changes in GRHL1 expression. It is also possible that, since miR-21–3p levels are relatively low in the normal HaCaT cell line (Fig. 3), the effect of miR-21–3p inhibition is much weaker and thus less evident than the effect of miR-21–3p overexpression. Although it is generally assumed that “passenger” strands of miRNAs are degraded upon miRNA processing, for several miRNAs it was shown that the -5p/−3p ratio varies depending on cell type, developmental stage or different disease states, what suggests that strand selection is a tightly controlled process. There is experimental evidence that miR-21–3p is present at high levels in the UV-induced epidermis enhancing inflammation that may facilitate or contribute to tumor growth [33]. It is thus possible that the expression of both *GRHL1* and *GRHL3* is regulated by the same oncogenic miRNA (miR-21), albeit by its different strands.

The oncogenic function of miR-21 is well documented. *miR-21*-deficient mice display decreased susceptibility to chemically-induced skin carcinogenesis [34]. The expression of miR-21-5p [35] and miR-21–3p [33] is induced by UV irradiation, a known risk factor in NMSC. Interestingly, miR-21 expression is suppressed by GRHL3, suggesting the existence of a regulatory loop [36]. The authors of that study proposed the following mechanism: upon malignant transformation the expression of *GRHL3* is reduced, which leads to increased expression of miR-21 and further enhancement of tumorigenesis. It would be tempting to speculate that, as a result of *GRHL3* decrease, the level of miR-21 increases which may subsequently lead to the reduction in the amount of *GRHL1* mRNA. However, in the light of a different publication this mechanism is unlikely, as *GRHL3* knock-down did not induce any changes in *GRHL1* expression in HaCaT cells [6]. It is thus more probable that the causes of reduced *GRHL1* and *GRHL3* expression in NMSC are environmental, occurring in response to UV irradiation, which triggers an increase in miR-21-5p and miR-21–3p levels in the epidermis, and in turn causes a decrease in the levels of *GRHL1* and *GRHL3* mRNA. Interestingly, in UVB-induced keratinocytes, there is a reduction of both transcript and protein level of desmoglein 1 [37], which is a direct target of GRHL1 regulation [4].

Our results shed further light on the importance of redundancy in studying carcinogenesis [1]. The functions of *Grhl1* and *Grhl3* genes are redundant in the maintenance of epidermal barrier in adult mice; both these genes have to be concomitantly inactivated in order for the impermeable skin barrier to be compromised [6]. Furthermore, it is known that barrier genes play important protective role in NMSC formation [11]. It is thus likely that, in the context of human NMSC, the expression of both *GRHL1* and *GRHL3* must be simultaneously reduced to delay epidermal cells differentiation and/or induce tumor promoting inflammatory microenvironment in the skin, which would enable skin tumor progression. This hypothesis is supported by our observation.

We did not detect any de novo mutations in any of the *GRHL* genes in NMSC samples, that is, mutations that would be present in tumors but absent from healthy tissue from the same patient. This result is consistent with earlier analyses of *GRHL3* gene in human SCC of the skin, where no de novo mutations were found [5]. It would be premature to speculate that *GRHL* genes do not undergo mutations in cancer, especially as they have been associated with many different types of cancer [8], but no such mutations have been reported to date.

We marked several polymorphisms with significantly altered frequencies in the NMSC patient cohort, in comparison with the European population (Table 1). Some of these variants are rare (allele frequency < 0.01). It is still a matter of debate whether rare or common variants are more likely to contribute to disease risk [38], hence we decided to include both types of alleles in our further analyses.

Non-synonymous SNP rs141193530 is located in exon 11 of the *GRHL3* gene and it introduces P455A amino acid residue substitution in the encoded GRHL3 protein. It seems likely that the presence of this SNP may be indeed deleterious for the functioning of the *GRHL3* gene, as this polymorphism is associated with two very different diseases: nsCL/P and NMSC ([32] and present study). However, in both these diseases the *GRHL3* gene serves a protective role. The reason why this SNP may be deleterious is related to the fact that the P455A substitution may nevertheless be damaging for the functioning of GRHL3 protein, despite some predictions to the contrary made by algorithms used by us and other researchers ([32] and present study). Alternatively, the presence of SNP rs141193530 may affect processing of the primary transcript, although our bioinformatic analyses did not provide any evidence to support this possibility. Thus the precise molecular mechanism responsible for the impact of SNP rs141193530 on the functioning of the *GRHL3* gene and/or the encoded GRHL3 protein remains to be elucidated.

Akin to the impairment of GRHL3 protein function or defective splicing of *GRHL3* pre-mRNA, reduced levels of *GRHL* gene expression in healthy human epidermis (prior to the development of NMSC) could also increase the risk of NMSC in affected people. This phenomenon is distinct from the reduction of expression of *GRHL1* and *GRHL3* genes in tumor samples in comparison with the adjacent healthy epidermis, as reduced *Grhl3* mRNA levels were observed in SCC samples in the *Grhl3*^+/+^ (wild-type) mice, which did not have any mutations or polymorphisms in the *Grhl3* gene [5]. Instead, it is related to the levels of expression of *GRHL* genes in histologically normal epidermis before the onset of NMSC. Previously it was shown that mouse models with significantly reduced epidermal expression levels of *Grhl1* or *Grhl3* display an increased susceptibility to SCC of the skin [5, 9]. In human subjects, such reduction in expression levels may be brought about by altered binding of transcription factors to the regulatory regions of *GRHL3* gene, which in turn may be caused by the presence of SNPs in binding motifs. Furthermore, the presence of SNPs in intronic regions may impact splicing efficiency, which may also lead to the reduction in mRNA levels (Table 1). These possibilities will require further studies.

## Conclusions

Presented results show that the growth of human non-melanoma skin cancers is accompanied by reduced levels of *GRHL1* and *GRHL3* mRNA. This decrease occurs in a coordinated manner, which suggests a common mechanism of regulation. It is possible that the expression of both genes is regulated, at least in part, by the same oncogenic microRNA: miR-21, which expression is induced by UV, the main causal factor of skin cancers formation. We did not find any de novo mutations in *GRHL* genes in human NMSC samples compared to adjacent normal skin. However, we observed that in the examined cohort of NMSC patients, some single nucleotide polymorphisms occur at significantly altered frequencies, in comparison with the general European population. The presence of these SNPs may affect the functioning of encoded proteins, splicing of primary transcripts, or may alter the binding of transcription factors to the regulatory regions of *GRHL* genes. Thus presence of these polymorphisms may indicate an altered risk of NMSC development in affected people.

## Additional files

Additional file 1: Table S1. Description of individuals with NMSC (fresh tissues, NGS). **Table S2.** SureSelect HaloPlex Design (analyzable regions). **Table S3.** Total number of NMSC patients including the replication cohort – general description. **Fig. S1.** Levels of GRHL1 protein in HaCaT cells transfected with miR-21–3p mimic or inhibitor. (DOC 240 kb)

## Abbreviations

BCC: Basal cell carcinoma
ENCODE: Encyclopedia of DNA Elements Consortium
ESE: Exonic splicing enhancer
ExAC: Exome aggregation consortium
FFPE: Formalin-fixed, paraffin-embedded
GRHL: Grainyhead-like
HDAC: Histone deacetylase
HPRT: Hypoxanthine-guanine phosphoribosyltransferase
NMSC: Non-melanoma skin cancer
nsCL/P: Nonsyndromic cleft lip with/without cleft palate
RFLP: Restriction fragment length polymorphism
SCC: Squamous cell carcinoma
SNP: Single nucleotide polymorphism
SRSF: Serine and arginine rich splicing factors
TBP: TATA box binding protein
TGM: Transglutaminase
UTR: Untranslated region
