# Supplementary material for: Coordinated expression and genetic polymorphisms in Grainyhead-like genes in human non-melanoma skin cancers
Source: BMC Cancer. 2018 Jan 4;18:23. doi: 10.1186/s12885-017-3943-8 (PMC5755140; doi:10.1186/s12885-017-3943-8)

**Table S1 Description of individuals with NMSC (fresh tissues, NGS)**

| Cancer type | Body part | | Age range |
| --- | --- | --- | --- |
| BCC | head and neck | cheek | 70-80 |
| BCC |  | head (skull) | 80-90 |
| SCC |  | forehead | 70-80 |
| SCC |  | cheek | 80-90 |
| BCC | front of trunk | abdomen | 50-60 |
| BCC |  | near collarbone | 70-80 |
| BCC |  | chest | 70-80 |
| BCC |  | near collarbone | 50-60 |
| SCC |  | near armpit | 60-70 |
| SCC |  | chest | 70-80 |
| BCC | back of trunk | shoulder blade | 80-90 |
| BCC |  | back | 60-70 |
| BCC |  | back | 80-90 |
| BCC |  | back | 70-80 |
| BCC |  | shoulder blade | 50-60 |
| BCC |  | shoulder | 70-80 |
| BCC |  | back | 50-60 |
| BCC |  | back | 20-30 |
| BCC |  | shoulder blade | 80-90 |
| BCC |  | shoulder blade | 60-70 |
| BCC |  | shoulder | 80-90 |
| SCC |  | back | 80-90 |
| BCC | upper limb | arm | 70-80 |
| SCC |  | hand | 70-80 |
| SCC |  | hand | 80-90 |
| SCC |  | arm | 60-70 |
| SCC |  | hand | 80-90 |
| BCC | lower limb | thigh | 90-100 |
| BCC |  | shank | 60-70 |
| BCC |  | shank | 60-70 |
| BCC |  | thigh | 80-90 |
| SCC |  | shank | 90-100 |
| SCC |  | thigh | 60-70 |

BCC basal cell carcinoma, SCC squamous cell carcinoma

**Table S2** SureSelect HaloPlex Design (analyzable regions)

| chromosome | gene | region |
| --- | --- | --- |
| chr1 | *GRHL3* | "NC_000001.10:24633720-24634608" |
| chr1 | *GRHL3* | "NC_000001.10:24651565-24651659" |
| chr1 | *GRHL3* | "NC_000001.10:24634614-24638115" |
| chr1 | *GRHL3* | "NC_000001.10:24638121-24651406" |
| chr1 | *GRHL3* | "NC_000001.10:24676886-24676980" |
| chr1 | *GRHL3* | "NC_000001.10:24673578-24673672" |
| chr1 | *GRHL3* | "NC_000001.10:24661370-24661464" |
| chr1 | *GRHL3* | "NC_000001.10:24666498-24666635" |
| chr1 | *GRHL3* | "NC_000001.10:24662808-24663150" |
| chr1 | *GRHL3* | "NC_000001.10:24671077-24671171" |
| chr1 | *GRHL3* | "NC_000001.10:24671780-24671874" |
| chr1 | *GRHL3* | "NC_000001.10:24671468-24671562" |
| chr1 | *GRHL3* | "NC_000001.10:24676343-24676792" |
| chr1 | *GRHL3* | "NC_000001.10:24663840-24664939" |
| chr1 | *GRHL3* | "NC_000001.10:24672749-24673362" |
| chr1 | *GRHL3* | "NC_000001.10:24660916-24661327" |
| chr1 | *GRHL3* | "NC_000001.10:24660799-24660893" |
| chr1 | *GRHL3* | "NC_000001.10:24663159-24663822" |
| chr1 | *GRHL3* | "NC_000001.10:24657564-24658360" |
| chr1 | *GRHL3* | "NC_000001.10:24671191-24671457" |
| chr1 | *GRHL3* | "NC_000001.10:24673804-24674183" |
| chr1 | *GRHL3* | "NC_000001.10:24668262-24669796" |
| chr1 | *GRHL3* | "NC_000001.10:24665849-24666478" |
| chr1 | *GRHL3* | "NC_000001.10:24681987-24682081" |
| chr1 | *GRHL3* | "NC_000001.10:24680534-24681947" |
| chr1 | *GRHL3* | "NC_000001.10:24690386-24690480" |
| chr1 | *GRHL3* | "NC_000001.10:24690514-24693364" |
| chr2 | *GRHL1* | "NC_000002.11:10080599-10080645" |
| chr2 | *GRHL1* | "NC_000002.11:10083354-10083448" |
| chr2 | *GRHL1* | "NC_000002.11:10094656-10094750" |
| chr2 | *GRHL1* | "NC_000002.11:10094871-10095427" |
| chr2 | *GRHL1* | "NC_000002.11:10084262-10084955" |
| chr2 | *GRHL1* | "NC_000002.11:10089711-10089835" |
| chr2 | *GRHL1* | "NC_000002.11:10089871-10090016" |
| chr2 | *GRHL1* | "NC_000002.11:10083458-10084248" |
| chr2 | *GRHL1* | "NC_000002.11:10092179-10092273" |
| chr2 | *GRHL1* | "NC_000002.11:10092047-10092169" |
| chr2 | *GRHL1* | "NC_000002.11:10079394-10080588" |
| chr2 | *GRHL1* | "NC_000002.11:10090021-10092038" |
| chr2 | *GRHL1* | "NC_000002.11:10084957-10086667" |
| chr2 | *GRHL1* | "NC_000002.11:10080653-10083350" |
| chr2 | *GRHL1* | "NC_000002.11:10088082-10089537" |
| chr2 | *GRHL1* | "NC_000002.11:10086678-10088072" |
| chr2 | *GRHL1* | "NC_000002.11:10098563-10098657" |
| chr2 | *GRHL1* | "NC_000002.11:10104710-10104804" |
| chr2 | *GRHL1* | "NC_000002.11:10100761-10100855" |
| chr2 | *GRHL1* | "NC_000002.11:10105030-10105124" |
| chr2 | *GRHL1* | "NC_000002.11:10102260-10102818" |
| chr2 | *GRHL1* | "NC_000002.11:10102860-10103023" |
| chr2 | *GRHL1* | "NC_000002.11:10099209-10099341" |
| chr2 | *GRHL1* | "NC_000002.11:10103835-10104653" |
| chr2 | *GRHL1* | "NC_000002.11:10098681-10099163" |
| chr2 | *GRHL1* | "NC_000002.11:10101899-10101993" |
| chr2 | *GRHL1* | "NC_000002.11:10105161-10105722" |
| chr2 | *GRHL1* | "NC_000002.11:10100901-10101780" |
| chr2 | *GRHL1* | "NC_000002.11:10125908-10126002" |
| chr2 | *GRHL1* | "NC_000002.11:10126144-10126602" |
| chr2 | *GRHL1* | "NC_000002.11:10135633-10135727" |
| chr2 | *GRHL1* | "NC_000002.11:10132949-10133043" |
| chr2 | *GRHL1* | "NC_000002.11:10140397-10140491" |
| chr2 | *GRHL1* | "NC_000002.11:10131094-10131188" |
| chr2 | *GRHL1* | "NC_000002.11:10138845-10138939" |
| chr2 | *GRHL1* | "NC_000002.11:10131772-10131866" |
| chr2 | *GRHL1* | "NC_000002.11:10133065-10133559" |
| chr2 | *GRHL1* | "NC_000002.11:10130569-10130663" |
| chr2 | *GRHL1* | "NC_000002.11:10130728-10131046" |
| chr2 | *GRHL1* | "NC_000002.11:10139078-10139406" |
| chr2 | *GRHL1* | "NC_000002.11:10140546-10142037" |
| chr2 | *GRHL1* | "NC_000002.11:10131870-10132546" |
| chr2 | *GRHL1* | "NC_000002.11:10135753-10136731" |
| chr2 | *GRHL1* | "NC_000002.11:10142042-10145498" |
| chr8 | *GRHL2* | "NC_000008.10:102505957-102506061" |
| chr8 | *GRHL2* | "NC_000008.10:102506064-102506244" |
| chr8 | *GRHL2* | "NC_000008.10:102495936-102496030" |
| chr8 | *GRHL2* | "NC_000008.10:102495523-102495925" |
| chr8 | *GRHL2* | "NC_000008.10:102494295-102495518" |
| chr8 | *GRHL2* | "NC_000008.10:102497793-102499029" |
| chr8 | *GRHL2* | "NC_000008.10:102519196-102519290" |
| chr8 | *GRHL2* | "NC_000008.10:102497386-102497790" |
| chr8 | *GRHL2* | "NC_000008.10:102508129-102508257" |
| chr8 | *GRHL2* | "NC_000008.10:102508363-102508562" |
| chr8 | *GRHL2* | "NC_000008.10:102500216-102501117" |
| chr8 | *GRHL2* | "NC_000008.10:102514552-102515329" |
| chr8 | *GRHL2* | "NC_000008.10:102493829-102494290" |
| chr8 | *GRHL2* | "NC_000008.10:102506246-102507277" |
| chr8 | *GRHL2* | "NC_000008.10:102496035-102497353" |
| chr8 | *GRHL2* | "NC_000008.10:102507288-102508089" |
| chr8 | *GRHL2* | "NC_000008.10:102501161-102502514" |
| chr8 | *GRHL2* | "NC_000008.10:102502671-102505946" |
| chr8 | *GRHL2* | "NC_000008.10:102508573-102511353" |
| chr8 | *GRHL2* | "NC_000008.10:102499040-102500207" |
| chr8 | *GRHL2* | "NC_000008.10:102515339-102515515" |
| chr8 | *GRHL2* | "NC_000008.10:102513008-102514541" |
| chr8 | *GRHL2* | "NC_000008.10:102511357-102513005" |
| chr8 | *GRHL2* | "NC_000008.10:102515524-102519186" |
| chr8 | *GRHL2* | "NC_000008.10:102555982-102556076" |
| chr8 | *GRHL2* | "NC_000008.10:102555204-102555903" |
| chr8 | *GRHL2* | "NC_000008.10:102565353-102565447" |
| chr8 | *GRHL2* | "NC_000008.10:102564665-102565247" |
| chr8 | *GRHL2* | "NC_000008.10:102564538-102564632" |
| chr8 | *GRHL2* | "NC_000008.10:102570263-102570357" |
| chr8 | *GRHL2* | "NC_000008.10:102571167-102571262" |
| chr8 | *GRHL2* | "NC_000008.10:102570407-102571163" |
| chr8 | *GRHL2* | "NC_000008.10:102582162-102582326" |
| chr8 | *GRHL2* | "NC_000008.10:102582339-102582924" |
| chr8 | *GRHL2* | "NC_000008.10:102585604-102586351" |
| chr8 | *GRHL2* | "NC_000008.10:102589199-102589293" |
| chr8 | *GRHL2* | "NC_000008.10:102589399-102590043" |
| chr8 | *GRHL2* | "NC_000008.10:102611056-102611716" |
| chr8 | *GRHL2* | "NC_000008.10:102631433-102631527" |
| chr8 | *GRHL2* | "NC_000008.10:102631582-102632148" |
| chr8 | *GRHL2* | "NC_000008.10:102643480-102643574" |
| chr8 | *GRHL2* | "NC_000008.10:102643950-102644147" |
| chr8 | *GRHL2* | "NC_000008.10:102643613-102643807" |
| chr8 | *GRHL2* | "NC_000008.10:102643818-102643945" |
| chr8 | *GRHL2* | "NC_000008.10:102644160-102644889" |
| chr8 | *GRHL2* | "NC_000008.10:102649484-102649578" |
| chr8 | *GRHL2* | "NC_000008.10:102648943-102649340" |
| chr8 | *GRHL2* | "NC_000008.10:102656738-102656832" |
| chr8 | *GRHL2* | "NC_000008.10:102656117-102656672" |
| chr8 | *GRHL2* | "NC_000008.10:102661982-102662133" |
| chr8 | *GRHL2* | "NC_000008.10:102661347-102661942" |
| chr8 | *GRHL2* | "NC_000008.10:102676985-102677079" |
| chr8 | *GRHL2* | "NC_000008.10:102676512-102676952" |
| chr8 | *GRHL2* | "NC_000008.10:102681849-102682122" |
| chr8 | *GRHL2* | "NC_000008.10:102682222-102682316" |
| chr8 | *GRHL2* | "NC_000008.10:102678772-102681822" |

**Table S3** Total number of NMSC patients including the replication cohort – general description

| patients | BCC | SCC | average age | median age |
| --- | --- | --- | --- | --- |
| NGS (fresh samples) | 22 | 11 | 72 | 76 |
| PyroSeq (FFPE samples) | 144 | 32 | 67 | 69 |
| Total | 166 | 43 | 68 | 69 |

**Fig S1** Levels of GRHL1 protein in HaCaT cells transfected with miR-21-3p mimic or inhibitor.

**A)** Level of GRHL1 protein in HaCaT cells transfected with miR-21-3p mimic. **B)** Level of GRHL1 protein in HaCaT cells transfected with miR-21-3p inhibitor. **C)** Control experiment: level of the *GRHL1* transcript in HaCaT cells transfected with miR-21-3p inhibitor.


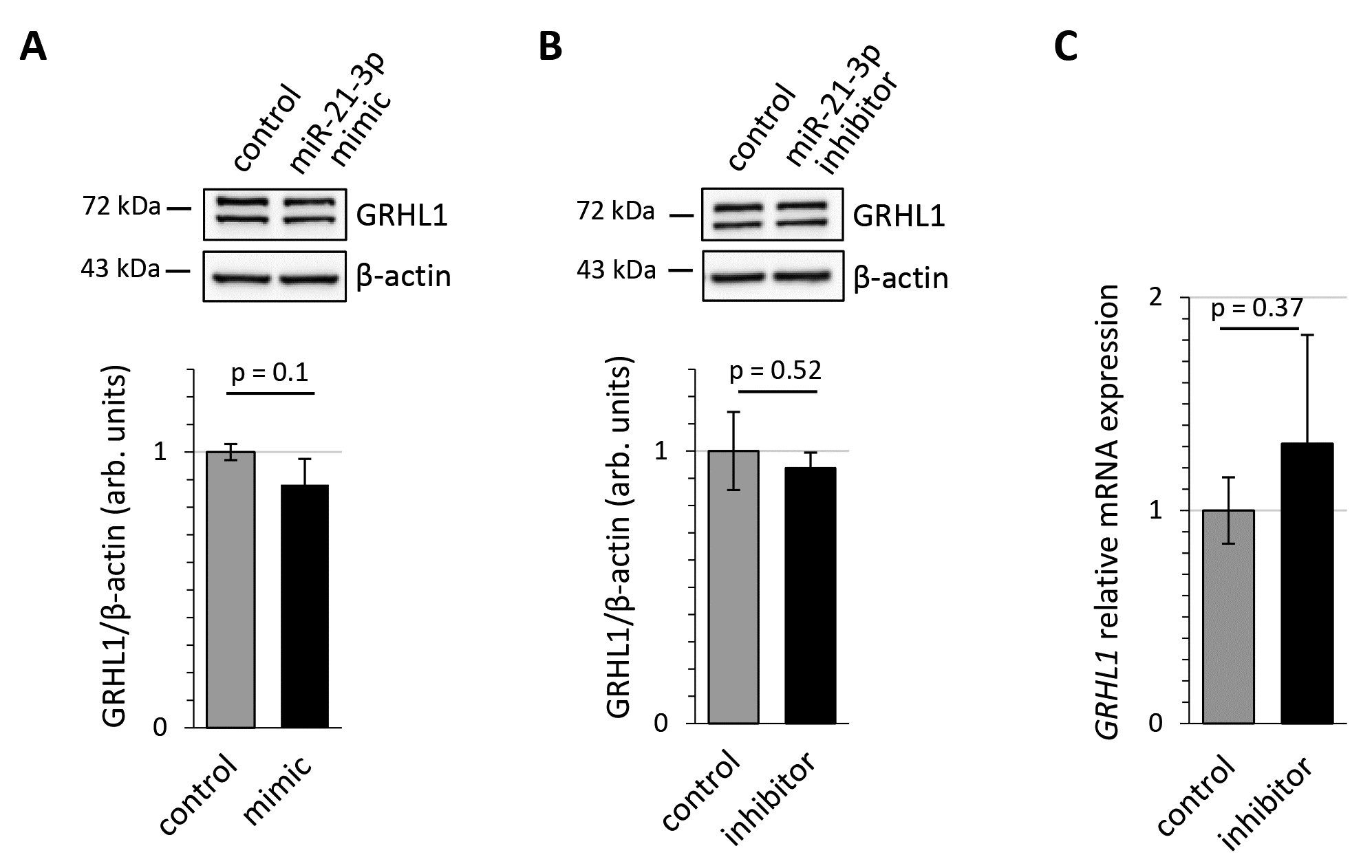

Supplement: Supplementary file 1 — Description of individuals with NMSC (fresh tissues, NGS). Table S2. SureSelect HaloPlex Design (analyzable regions). Table S3. Total number of NMSC patients including the replication cohort – general description. Fig. S1. Levels of GRHL1 protein in HaCaT cells transfected with miR-21–3p mimic or inhibitor. (DOC 240 kb) [file 12885_2017_3943_MOESM1_ESM.doc]
